# Osteoid Osteoma at the Lesser Trochanter: A Lesson in Mimicry

**DOI:** 10.7759/cureus.5425

**Published:** 2019-08-19

**Authors:** Andrew S Murtha, Nathan D Cecava, Dustin O Lybeck

**Affiliations:** 1 Orthopaedic Surgery, Brooke Army Medical Center, Fort Sam Houston, USA; 2 Radiology, Wilford Hall Ambulatory Surgical Center, San Antonio, USA

**Keywords:** osteoid osteoma, adolescent athlete, cryoablation, hip

## Abstract

A 16-year-old female soccer player presented with an eight-month-old, insidious right-hip pain. The imaging workup demonstrated a cortically based lytic lesion at the level of the lesser trochanter with surrounding sclerosis, adjacent periosteal reaction, and a small soft-tissue mass. A biopsy revealed findings related to an osteoid-forming lesion with features of nuclear atypia. The patient's pathology referral returned a diagnosis of osteoid osteoma, and she was treated with CT-guided cryoablation. She had an excellent response and returned to full activities through nearly two years of surveillance. In similar patients approaching skeletal maturity, the differential diagnosis for bone lesions involving the lesser trochanter ranges from post-traumatic to primary oncologic processes. Given the implications of a missed malignancy, vigilance is required when treating young and active patients.

## Introduction

Osteoid osteoma is a common benign bone lesion found in adolescents. It accounts for 10-12% of benign bone tumors and usually affects the long bones of the lower extremity, with two-thirds of the femoral lesions occurring in the pertrochanteric region [1-6]. Multiple series have demonstrated a male predominance, with Kransdorf et al. noting a male-to-female ratio of 3.85:1 in a series from the Armed Forces Institute of Pathology in Washington, D.C. [7]. Classic findings in a patient’s history include an insidious onset of pain, often at night, which responds to non-steroidal anti-inflammatory medications (NSAIDs). Radiographs typically reveal an eccentric or juxta-cortical lesion less than 2 cm in diameter with surrounding sclerosis and periosteal reaction [1, 3-4]. CTs often show a radiolucent nidus surrounded by reactive sclerosis. The nidus can sometimes contain a sclerotic center [6]. MRIs typically demonstrate a profound degree of perilesional bone edema without an associated soft-tissue mass [1, 4]. Treatment of these lesions depends on their location and consists of symptomatic management with NSAIDs, radiofrequency ablation, cryoablation, or en bloc resection [1, 3, 8].

In skeletally immature athletes, apophyseal avulsions of the anterior superior iliac spine (ASIS), anterior inferior iliac spine (AIIS), ischial tuberosity, or lesser trochanter can result from sudden, forceful contractions of originating muscle groups [2, 9-12]. These injuries may heal with robust callus formation that can alter the femoral or pelvic morphology and mimic neoplastic processes [13]. As a secondary ossification center of the proximal femur, the lesser trochanter typically appears around the age of 11 years in females and the age of 12 years in males, with closure seen around the age of 16-17 years [13-14]. In skeletally mature patients, avulsions of the lesser trochanter are considered pathognomonic of a malignant process in the proximal femur [15-17].

In adolescent athletes, an osteoid osteoma at the lesser trochanter may mimic an apophyseal avulsion fracture or an osteogenic sarcoma. We present the case of a 16-year-old female with such a presentation and discuss the diagnostic challenges. The patient and her family have given their consent for the publication of this case.

## Case presentation

**Figure 1:**
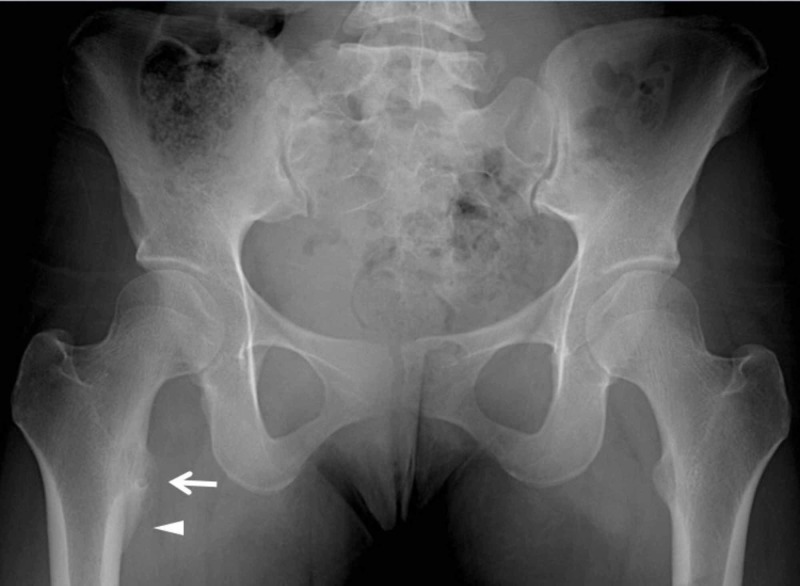
Anterior-posterior radiograph of the pelvis obtained five months after the symptom onset. A 16-year-old female with a lytic lesion of the right femoral lesser trochanter cortex. Anterior-posterior radiograph of the pelvis obtained five months after the symptom onset shows a lytic lesion (arrow) with indistinct margins and no definite nidus. There is associated smooth periosteal reaction (arrowhead).

A 16-year-old female presented with an eight-month old right-hip pain of insidious onset. She was an avid high-school soccer player but was unable to attribute the pain to a specific incident. She had initially consulted her primary care physician (PCP) after two months of symptoms and had been prescribed a 10-week course of physical therapy. When her symptoms failed to resolve and the pain was noted to further localize to the hip and buttock, plain radiographs were obtained. They demonstrated a cortically based lytic lesion at the level of the lesser trochanter with surrounding sclerosis and adjacent periosteal reaction (Figure 1).

**Figure 2:**
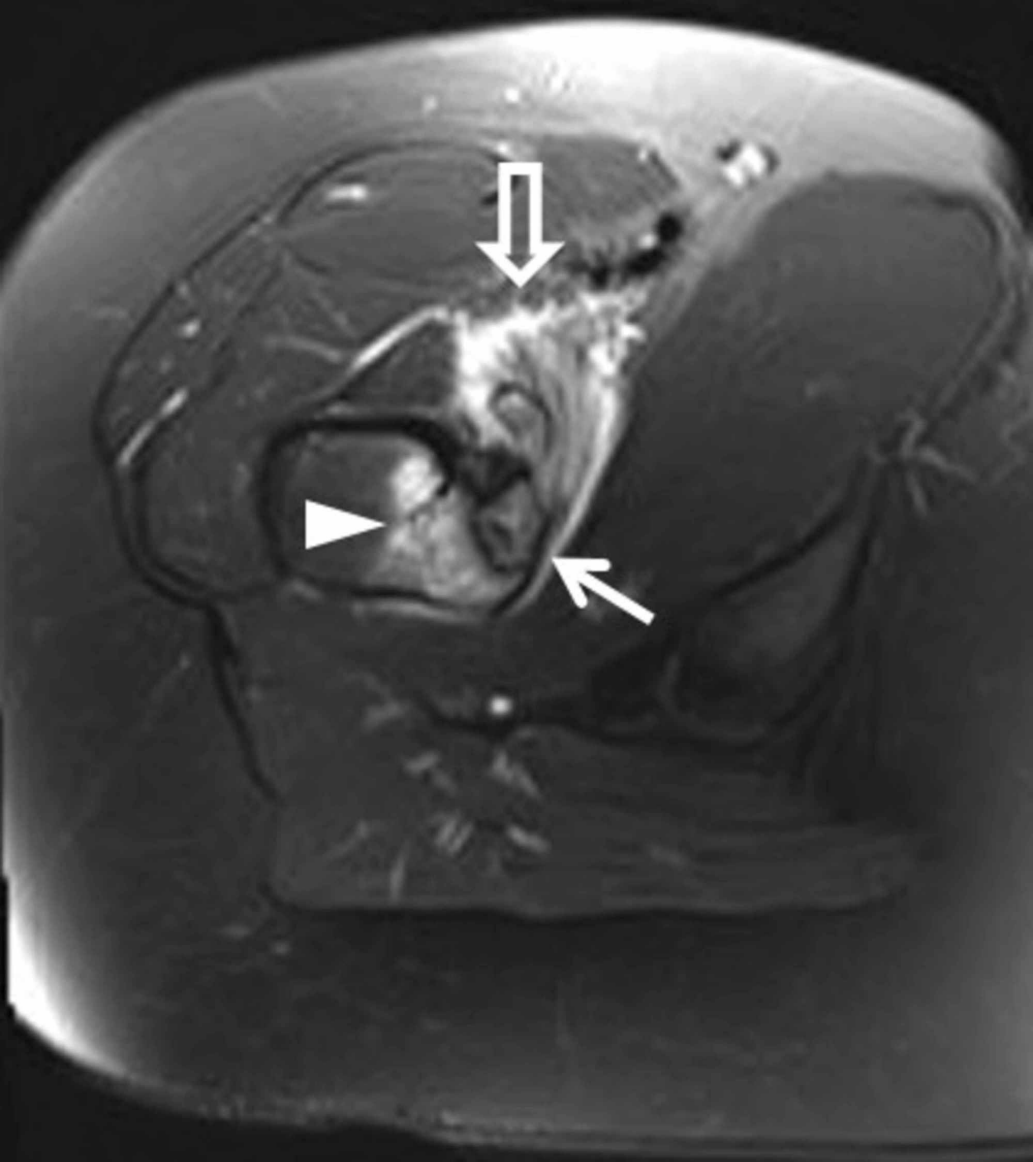
MR axial T2 fat saturation image obtained seven months after symptom onset. A 16-year-old female with a lytic lesion of the right femoral lesser trochanter cortex. MR axial T2 fat saturation image obtained seven months after symptom onset. The cortically based lesion with discontinuous sclerotic rim (closed arrow) has T2 hyperintense signal. There is associated periosteal edema and bone marrow edema (arrowhead). Enhancing iliopsoas bursitis is also present (open arrow). The lesion had intermediate T1 signal (not shown).

**Figure 3:**
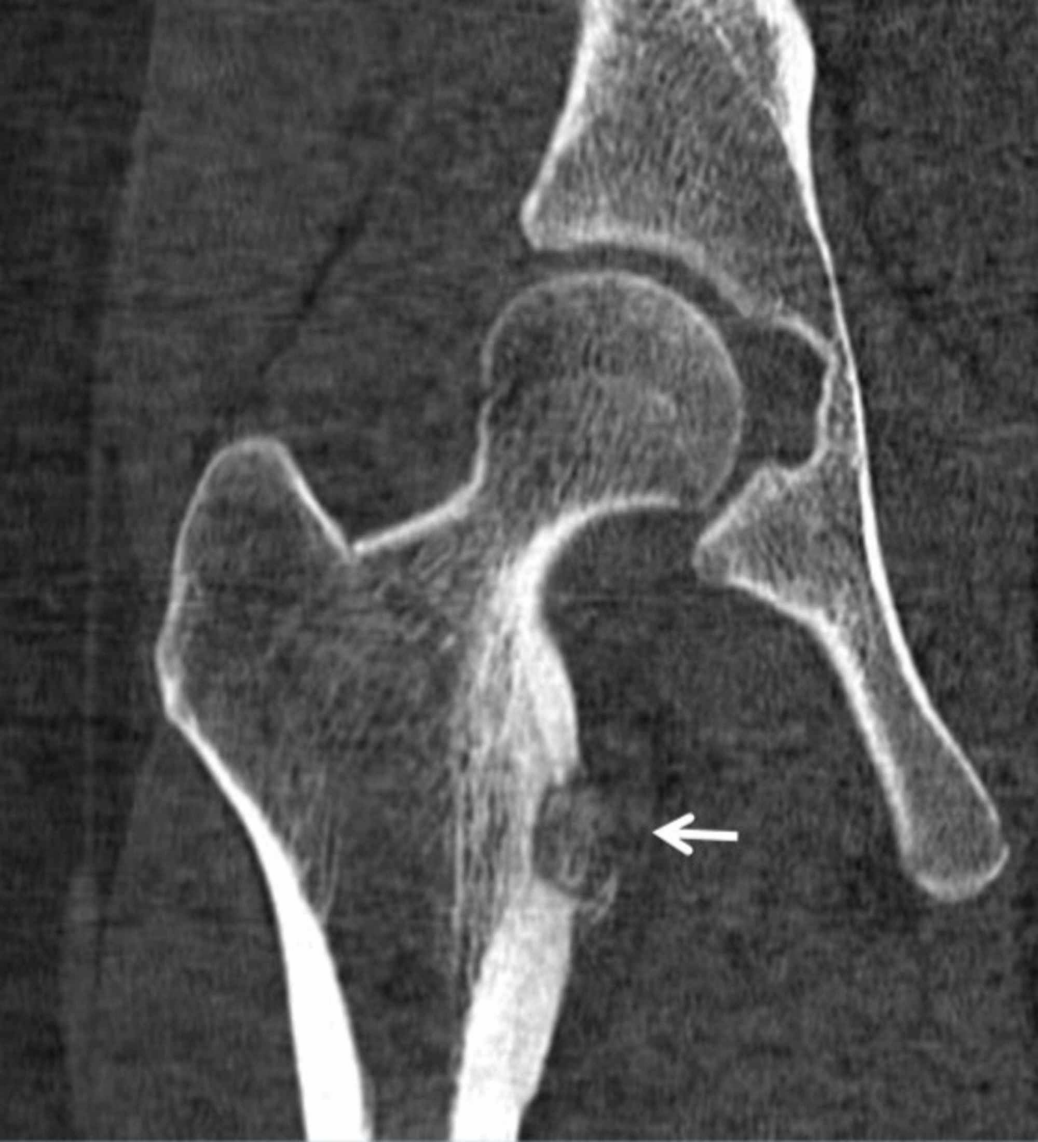
CT image obtained seven months after symptom onset. A 16-year-old female with lytic lesion of the right femoral lesser trochanter cortex. Coronal CT image obtained seven months after symptom onset shows a cortically based lytic lesion (arrow) with adjacent periosteal reaction and adjacent reactive sclerosis. No presence of an internal sclerotic nidus. The lesion has internal osteoid matrix with extra osseous matrix extension in the medial soft tissues at the iliopsoas tendon insertion.

Advanced imaging was then ordered, and the patient was referred to a local orthopedic surgeon. After evaluation, her care was transferred to an orthopedic oncologist. On presentation, her pain was noted to be worse at night and slightly alleviated by naproxen. She had pain with resisted hip flexion, but her exam was otherwise benign. An MRI with intravenous contrast was obtained (Figure 2), along with a CT (Figure 3), and a scintigraphic bone scan.

Although the patient’s history and exam were suggestive of a benign or post-traumatic etiology, the findings of an adjacent periosteal reaction on plain radiographs (Figure 1) and a soft-tissue mass with areas of ossification on CT (Figure 3) were not entirely exclusive of a primary bone sarcoma. Hence, the patient was referred for a CT-guided core bone biopsy.

**Figure 4:**
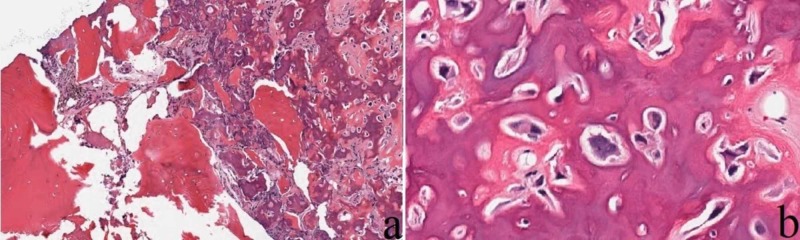
Core needle biopsy. A 16-year-old female with lytic lesion of the right femoral lesser trochanter cortex. Core needle biopsy demonstrates a well-circumscribed lesion (a, H&E, original magnification 40x), with woven bone and osteoblasts demonstrating features of nuclear atypia (b, H&E, original magnification 400x).

Initial review of the biopsy demonstrated findings regarding an osteoid-forming lesion with features of nuclear atypia (Figure 4).

**Figure 5:**
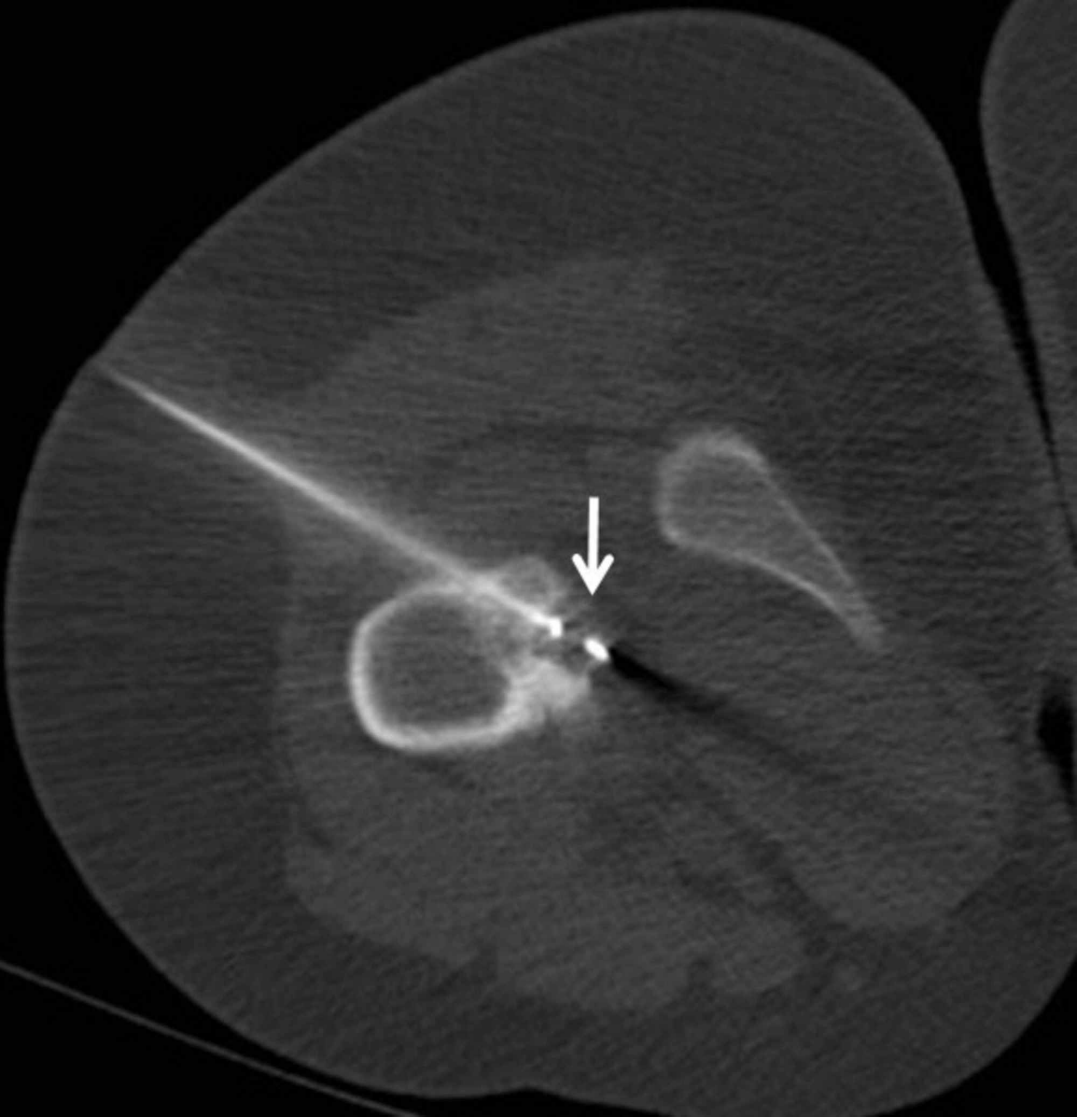
Axial CT image shows CT-guided placement of a cryotherapy probe. A 16-year-old female with lytic lesion of the right femoral lesser trochanter cortex. Axial CT shows CT-guided placement of a cryotherapy probe into the lesion from posterolateral approach.

Lesions in the differential included osteoid osteoma, osteoblastoma, osteosarcoma, and callus reaction from an avulsion fracture. These findings were corroborated by a consulting pathologist. The pathologist's opinion favored fracture and close follow-up or a repeat biopsy was recommended. A third opinion was sought from a national pathology referral center. Finally, combining history, radiographic differential, and histologic appearance, the diagnosis of osteoid osteoma was made. The patient was treated with CT-guided cryoablation (Figure 5).

**Figure 6:**
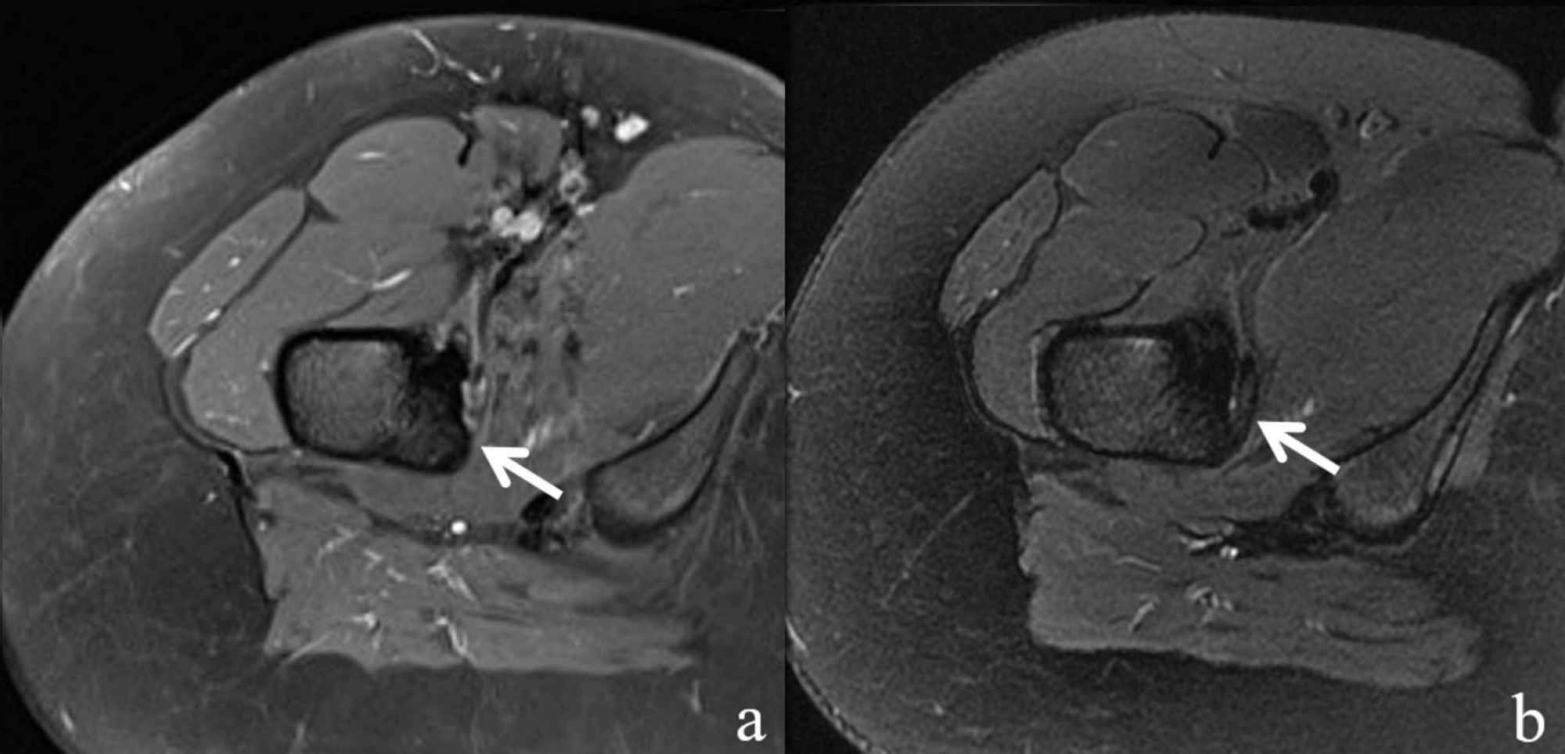
MR axial T2 fat saturation images after treatment. A 16-year-old female with lytic lesion of the right femoral lesser trochanter cortex. MR axial T2 fat saturation image obtained five months after treatment (a) and 21 months after treatment (b). Lesion signal has become isointense to the surrounding bone marrow with restoration of the cortex (arrows). Periosteal edema, bone marrow edema, and iliopsoas bursitis have resolved.

She recovered from this procedure with a resolution of her symptoms and has returned to participation in high-school athletics. Surveillance radiographs and MRI were obtained after five months and 21 months following her ablation, and they all demonstrated resolution of the adjacent periosteal edema, bone marrow edema, and iliopsoas bursitis with restoration of the cortex (Figure 6).

## Discussion

The proximal femur is a common site for the development of an osteoid osteoma. In patients approaching skeletal maturity, the differential for lesions involving the lesser trochanter ranges from post-traumatic to primary oncologic processes. Despite a history suggestive of a benign process in the case under discussion, the imaging workup was equivocal. And the results of the biopsy only further confounded the treatment team as features of nuclear atypia, hypercellularity, and disorganized growth could be seen in both fracture callus and osteogenic malignancies [18]. 

Osteoid osteoma is most frequently seen in adolescents and young adults. Most cases were reported in patients between the ages of 5-24 years [1, 4-5]. Within long bones, these benign tumors are typically situated within the diaphysis or metaphysis, and they have been further subtyped according to their location within the bone as cortical (75%), medullary (20%), or subperiosteal (5%) [4, 6]. Cortical lesions in diaphyseal locations typically demonstrate the classic nidus with abundant reactive sclerosis. Lesions in other locations demonstrate comparatively little to no sclerosis [4, 6]. Medullary and subperiosteal lesions may also occur in intraarticular or periarticular locations, such as the femoral neck, and cause profound reactive synovitis [2, 4-6].

In skeletally immature athletes, the physis or apophysis is weaker than the adjacent attachments of ligaments and tendons. As such, injuries that might cause sprains, strains, or ruptures of ligaments or tendons in adults often lead to physeal or apophyseal injuries in children [13]. Due to the innate healing capacity of physeal tissues, exuberant callus formation is often seen following such injuries. In activities that require forceful hip flexion, such as soccer or track, avulsions of the iliopsoas insertion on the lesser trochanter can lead to an alteration of the proximal femoral morphology secondary to the resulting callus [19]. Given the inherent resilience of most young patients, such injuries may not be recognized until months later when substantial healing has already occurred. In such circumstances, the diagnosis is also confounded by the knowledge that many young patients with benign and malignant bone lesions often present with indolent pain and correlate it with some form of minor trauma [2, 5, 20].

## Conclusions

This difficult case required clinical correlation, an expert opinion from an experienced pathologist, and an interdisciplinary team including orthopedic oncology and musculoskeletal radiology to ensure proper diagnosis and treatment. Given the profound implications of a misdiagnosis, vigilance is required when treating young and active patients.
